# The effect of fear of missing out on mental health: differences in different solitude behaviors

**DOI:** 10.1186/s40359-023-01184-5

**Published:** 2023-05-01

**Authors:** Xinyang Liu, Tour Liu, Zhao Zhou, Fuyu Wan

**Affiliations:** 1 Faculty of Psychology, Tianjin Normal University, Tianjin, China; 2 Key Research Base of Humanities and Social Sciences of the Ministry of Education, Academy of Psychology and Behavior, Tianjin Normal University, Tianjin, China; 3 Tianjin Social Science Laboratory of Students’ Mental Development and Learning, Tianjin, China

**Keywords:** Fear of missing out, Mental health, Solitude behavior, Social avoidance, Loneliness

## Abstract

**Background:**

Depression, anxiety, and stress are the main issues that affect the mental health of individuals. Solitude behavior, fear of missing out, and mental health are all closely related.

**Objective:**

This study was intended to investigate the relationship between solitude behavior, fear of missing out, and mental health.

**Methods:**

Short Form of Solitude Behavior Scale, Fear of Missing Out scale, and Depression Anxiety Stress Scale-21 were employed in this study to investigate 616 college students. The collected data were analyzed using SPSS 26.0 for basic data organization, and Mplus 8.3 was used to complete the analysis of the mediation model.

**Results:**

(1) Positive solitude was positively associated with eccentricity and negatively related to loneliness; social avoidance positively correlated with eccentricity and loneliness. (2) Social avoidance and loneliness affected mental health through the mediating effect of fear of missing out, whereas positive solitude and eccentricity did not affect mental health through fear of missing out. Moreover, the results still held in the model with depression, anxiety, and stress as dependent variables.

**Conclusion:**

The roles of different solitude behavior in the relationship between fear of missing out and mental health differed. Social avoidance and loneliness as not self-determined solitude could activate the fear of missing out, which could affect mental health.

## Introduction

Anxiety, depression, stress, and other mental health issues have gradually become more prominent due to society’s accelerated pace and fiercer competition in various fields. A survey showed that 14.97% of Chinese adults suffered the high-risk psychological disorders [1]. In recent years, mental health issues have caught much attention and become a long focus in psychological research. Poor mental health can cause a series of effects, such as physical health problems, interpersonal problems, cognitive states, and even extreme behaviors such as suicide [2–5]. Therefore, researchers have concentrated on figuring out the psychological mechanisms underlying mental health issues including depression, anxiety, and stress, as well as their causes. This has helped to advance the creation of effective therapeutic strategies.

A robust impact on mental health by fear of missing out (FoMO) was verified by many researchers recently [6, 7]. FoMO is a diffuse anxiety that occurs when individuals fear missing out on the positive experiences of others [8, 9]. Numerous research has discovered a connection between mental health and FoMO. For example, FoMO was positively correlated with depression and anxiety [6, 10] and significantly predicted stress [11]. Individuals with high levels of FoMO were more likely to suffer from alcohol abuse [12] and sleep disorders [13]. FoMO also decreased well-being [14] and life satisfaction [9]. Overall, existing research consistently shows that FoMO has a negative impact on mental health.

With the deepening of FoMO research, solitude behavior as a strong predictor of FoMO was discussed [15]. Chen et al. proposed that solitude behavior refers to a state in which individuals have no information and emotional communication with others when they are conscious [16]. Dhir et al. argued that individuals with FoMO always expect to be aware of the activities of others on a continuous basis [17]. It can be assumed that individuals in solitude are more likely to have a high FoMO. This has been confirmed by previous research, with Lai et al. finding that individuals with higher levels of loneliness had more severe FoMO on important information [18]. Bernard and Barry & Wong both found a significant relationship between loneliness and FoMO [13, 19]. Cheng also pointed out that loneliness had a direct positive predictive effect on the FoMO in an adolescent study [20]. Thus, this means that solitude behavior may activate the FoMO. Combined with the fact that FoMO negatively affects mental health, it was logical to assume that there may be a mediating mechanism from solitude behavior to FoMO and then to mental health.

Although existing studies have shown an association between solitude and FoMO, a review of existing research reveals that the increased FoMO in solitary individuals is primarily associated with increased feelings of loneliness. The motivation for solitude as an episodic behavior may vary widely. Nicol divided solitude behavior into self-determined solitude and non-self-determined solitude [21]. Chen et al. suggested that solitude behavior can include four types of behaviors resulting from different causes or motivations, namely positive solitude, eccentricity, social avoidance, and loneliness [16]. Positive solitude refers to the ability to enjoy independent activities. Eccentricity is a personality trait in which people feel comfortable being alone and usually fail to maintain good interpersonal relationships with others. Differing from positive solitude, eccentricity is sometimes accompanied by passive solitude. For social avoidance, interpersonal scenarios make individuals feel fearful and anxious, so they choose to avoid social situations to alleviate negative emotions. Loneliness is a subjective experience that arises when interpersonal needs cannot be met and often produces negative experiences when alone. Positive solitude and eccentricity are self-determined, whereas social avoidance and loneliness are non-self-determined. Lu et al. found that individuals could have four solitude behaviors at the same time, only the high and low levels of the four solitudes differ [22]. It was evident that solitude behaviors have complex motivational components, and the relationship between these complex components and fear of missing out and mental health remains unclear.

According to the *Self-Determination Theory*, the healthy development of individuals depended on the satisfaction of three basic needs: autonomy needs, competence needs, and relatedness needs. Among them, Relatedness needs refer to those needs that are satisfied by establishing social relationships with others [23]. FoMO occurred when an individual’s interpersonal needs were not met [8, 24]. The study found that frequent use of social media kept people in a constant state of connectedness, which in turn increased the FoMO [24]. Milyavskaya et al. noted that FoMO that arose when individuals carried out activities with other people was lower than individuals interacted with their natural environment [11]. FoMO may increase if association with others cannot be maintained. Accordingly, this study hypothesized that individuals who exhibited solitude may have higher levels of FoMO when non-self-determined solitude was dominant, and conversely no association with FoMO when self-determined solitude was dominant.

In summary, this study intended to investigate three main questions: (1) how solitude behavior was related to FoMO and mental health; (2) whether FoMO played a mediating role in the relationship between solitude behavior and mental health; and (3) whether there were differences in the effects of the four different types of solitude on mental health through FoMO.

## Method

### Participants and procedure

This study recruited 616 college and graduate students from a university in Tianjin, China, to complete questionnaires. Convenience sampling was conducted. The mean age of the study population was 19.82 years (SD = 2.23), with 114 males and 494 females (eight participants did not report their gender). There were 273 (44.3%) freshmen, 144 (23.4%) sophomores, 56 (9.1%) junior students, 21 (3.4%) senior students, 112 (18.2%) first-year graduate students, 4 other grades, and six participants did not report their grade. The estimated sample size to detect a mediated effect with power of 0.80 is estimated to be 462 [25]. This study included 616 participants and it could be sufficient to detect the predicted effects. The collected data were analyzed using SPSS 26.0 for basic data organization, and Mplus 8.3 was used to complete the analysis of the mediation model. Pearson correlation was conducted to examine the correlations between all main variables. The primary analysis of the mediation model method was path analysis with maximum likelihood (ML) estimation. The bootstrap method, which repeatedly draws random samples from the original data with replacement, was used to evaluate the mediation effect. We used 5000 bootstrap resamples for this analysis to compute the 95% confidence intervals. Confidence intervals were then tested for significance by examining whether or not they contained zero.

### Measurement

*The Short Form of Solitude Behavior Scale* (SBS-S) was used to assess participants’ solitude behavior in a 16-item scale consisting of four dimensions: positive solitude, eccentricity, social avoidance, and loneliness [26]. Each dimension has 4 items and is rated on a 5-point Likert scale (1 “strongly disagree” to 5 “strongly agree”). Some example items were “I sometimes like to read and think alone”, “I like to be alone and have little interest in other people”, “I usually feel uncomfortable when I’m with a group of people I don’t know”, “I feel lonely when no one is with me”. The Cronbach *α*s of the scale in this study was 0.81, and the Cronbach *αs* of each dimension ranged from 0.82 to 0.88.

*The fear of Missing Out scale* (FoMOs) was used to assess participants’ FoMO [9]. This is a 10-item scale containing only one dimension on a 5-point scale (1 “strongly disagree” to 5 “strongly agree”). One example item was “I fear others have more rewarding experiences than me”. The Cronbach *α* of the scale in this study was 0.79.

*The Depression Anxiety Stress Scale-21* (DASS-21) was used to assess participants’ mental health in a 21-item scale consisting of three dimensions: depression, anxiety, and stress [27]. Each dimension has 7 items and is rated on a 4-point Likert scale (1 “extremely unlikely” to 4 “always meet”). Some example items were “I was aware of dryness of my mouth”, “I found it hard to wind down”, and “I couldn’t experience positive feeling”. The Cronbach αs of the scale in this study was 0.93, and the Cronbach αs of each dimension ranged from 0.78 to 0.87.

## Result

### Correlation analysis result among all variable

In this study, the data were tested for common method variance using *Harman’s Single-Factor Test*, and there were nine factors with an eigenvalue greater than 1, and the first factor only accounted for 26.07% (less than 40% of the total variation), so there was no significant CMV exists.

According to the results generated from the correlation shown in Table 1. Positive relationships were found between FoMO and mental health, depression, anxiety, and stress. Similarly, FoMO was significantly and positively related to solitude behavior, social avoidance, and loneliness. In addition, the correlation between positive solitude and eccentricity (*r* = 0.20) was significant; the correlation between social avoidance and loneliness (*r* = 0.45) was significant.

**Table 1:** Descriptive statistics and correlations for the study variables

	$\stackrel{-}{x}$±*s*	1	2	3	4	5	6	7	8	9
1 Positive solitude	17.29 ± 2.64	-	-	-	-	-	-	-	-	-
2 Eccentricity	9.98 ± 3.73	0.20^***^	-	-	-	-	-	-	-	-
3 Social avoidance	13.22 ± 4.17	0.06	0.33^***^	-	-	-	-	-	-	-
4 Loneliness	11.05 ± 4.00	-0.21^***^	0.03	0.45^***^	-	-	-	-	-	-
5 Solitude behavior	51.58 ± 8.98	0.31^***^	0.63^***^	0.81^***^	0.60^***^	-	-	-	-	-
6 FoMO	30.14 ± 6.81	-0.06	0.07	0.38^***^	0.57^***^	0.43^***^	-	-	-	-
7 Depression	11.78 ± 3.63	-0.09^*^	0.30^***^	0.35^***^	0.45^***^	0.45^***^	0.41^***^	-	-	-
8 Anxiety	11.89 ± 4.31	-0.05	0.31^***^	0.43^***^	0.45^***^	0.51^***^	0.44^***^	0.77^***^	-	-
9 Stress	10.36 ± 3.65	-0.09^*^	0.37^***^	0.36^***^	0.41^***^	0.47^***^	0.34^***^	0.71^***^	0.82^***^	-
10 Mental health	33.99 ± 10.58	-0.08	0.36^***^	0.42^***^	0.46^***^	0.52^***^	0.43^***^	0.90^***^	0.95^***^	0.91^***^

FoMO: fear of missing out. $\stackrel{-}{x}$: mean, *s*: standard deviation. ^*^p < 0.05, ^**^p < 0.01, ^***^p < 0.001.

Correlations are Pearson correlations.

### The role of solitude behavior in the relationship between FoMO and mental health

**Fig. 1:**
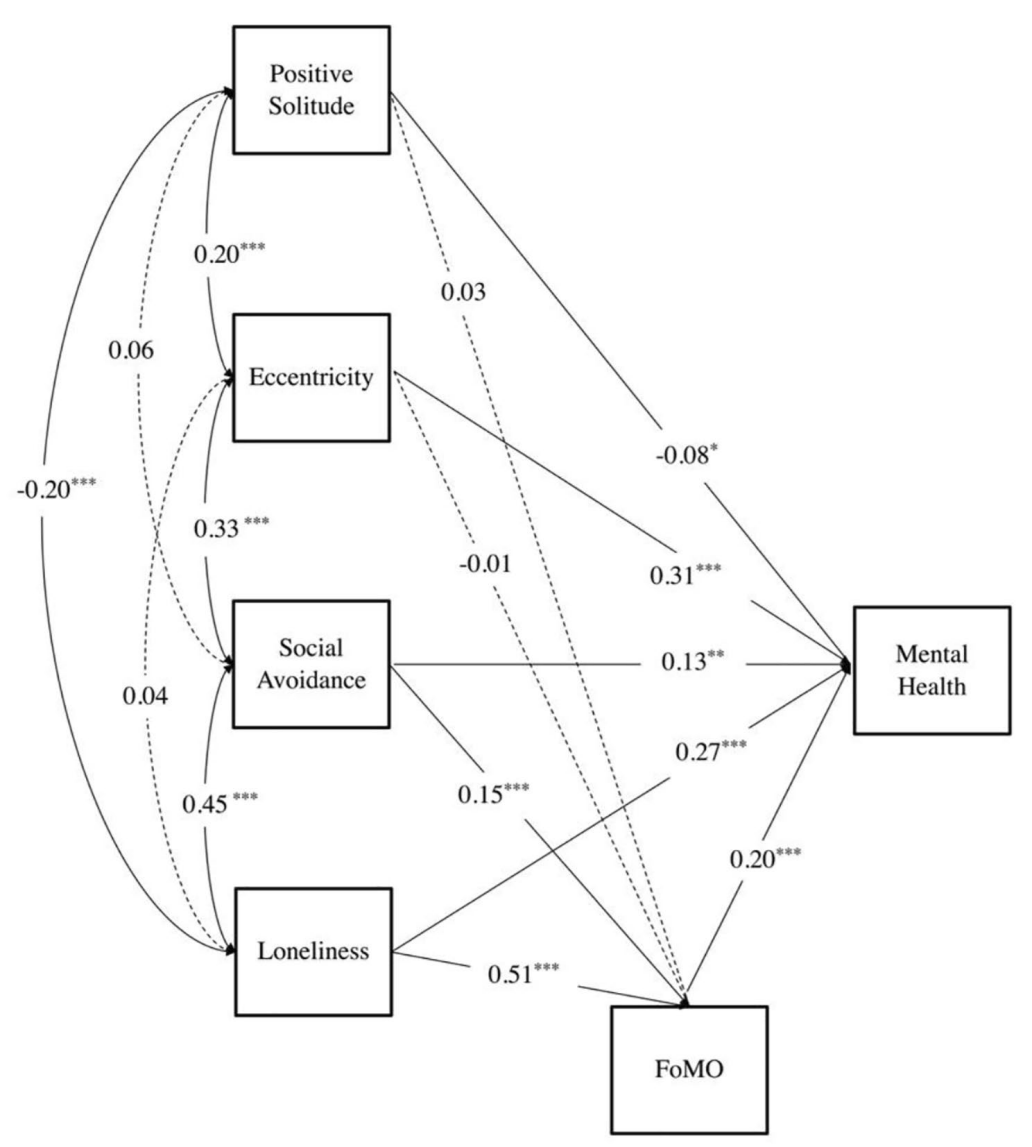
The Role of Solitude Behavior in the Relationship between FoMO and Mental Health *The dashed line indicated that the path coefficient was not significant, and the solid line indicated that the path coefficient was significant*.

Based on the hypotheses of this study, we constructed mediation models using positive solitude, eccentricity, social avoidance, and loneliness as independent variables, FoMO as mediating variables, and mental health as the dependent variable, and the model results were shown in Fig. 1. Since the mediation models in this study were all saturated models, no further model fit indicators were reported. The results indicated that positive solitude negatively predicted mental health; while eccentricity, social avoidance, loneliness, and FoMO positively predicted mental health. In addition, social avoidance and loneliness were significant predictors of FoMO.

The Bootstrap method with 5000 samples was used to examine the mediation effects, and the results showed that the mediation effects of social avoidance and loneliness in predicting mental health through FoMO accounted for 19.23% and 28.10% of the total effect, respectively, and the 95% confidence interval for each mediating pathway did not contain 0. While positive solitude and eccentricity could not predict mental health through FoMO (see Table 2).

**Table 2:** The mediation effect of FoMO between solitude behaviors and mental health

Path	*β*	*SE*	Bootstrap 95% CI		Effect Size
			Boot LLCI	Boot ULCI	
Positive Solitude → FoMO → Mental Health	0.01	0.01	-0.01	0.02	-
Eccentricity → FoMO → Mental Health	0.00	0.01	-0.02	0.02	-
Social Avoidance → FoMO → Mental Health	0.03^**^	0.01	0.01	0.05	19.23%
Loneliness → FoMO → Mental Health	0.10^***^	0.02	0.06	0.15	28.10%

FoMO: fear of missing out. *β*: standardized indirect effects, *SE*: standard error.

^*^p < 0.05, ^**^p < 0.01, ^***^p < 0.001.

Bootstrap method with 5000 bootstrap samples is used to compute the 95% confidence intervals.

### The role of solitude behavior in the relationship between FoMO and depression anxiety, and stress

**Fig. 2:**
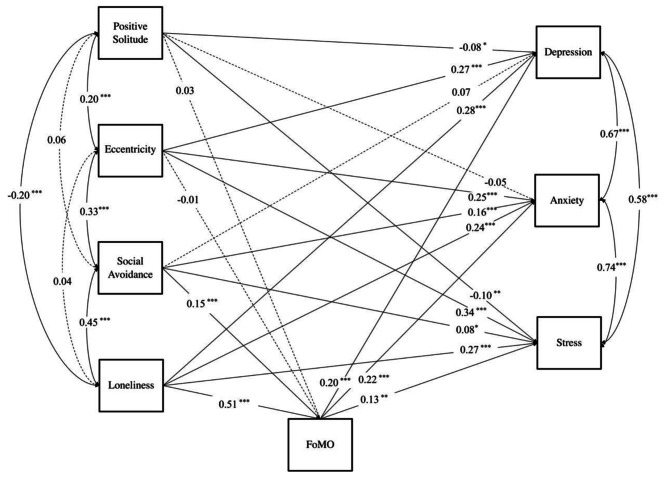
The Role of Solitude Behavior in the Relationship between FoMO and Depression, Anxiety, and Stress

The results of the above mediation model suggested that different solitude behaviors play different roles in the relationship between FoMO and mental health. To further understand the role of solitude behaviors and FoMO in different types of mental health problems, this study analyzed depression, anxiety, and stress as dependent variables, and the results of the model were shown in Fig. 2. The results showed that positive solitude negatively predicted depression and stress; social avoidance positively predicted anxiety and stress. Eccentricity, loneliness, and FoMO all positively predicted depression, anxiety, and stress. In addition, consistent with the results obtained previously, FoMO was positively associated with social avoidance and loneliness.

The Bootstrap method with 5000 samples was used to examine the mediation effects, and the results showed that the mediation effects of social avoidance and loneliness in predicting depression through FoMO accounted for 30.93% and 27.01% of the total effect, in predicting anxiety through FoMO accounted for 16.84% and 32.11% of the total effect, in predicting stress through FoMO accounted for 18.81% and 20.06% of the total effect. The 95% confidence interval for each mediated pathway did not contain 0. And positive solitude and eccentricity did not predict depression, anxiety, and stress through FoMO (see Table 3).

**Table 3:** The mediation effect of FoMO between solitude behaviors and depression, anxiety, and stress

Path	*β*	*SE*	Bootstrap 95% CI		Effect Size
			Boot LLCI	Boot ULCI	
Positive Solitude → FoMO → Depression	0.01	0.01	-0.01	0.02	-
Eccentricity → FoMO → Depression	0.00	0.01	-0.02	0.02	-
Social Avoidance → FoMO → Depression	0.03^**^	0.01	0.01	0.06	30.93%
Loneliness →FoMO → Depression	0.10^***^	0.03	0.06	0.16	27.01%
Positive Solitude → FoMO → Anxiety	0.01	0.01	-0.01	0.03	-
Eccentricity → FoMO → Anxiety	0.00	0.01	-0.02	0.02	-
Social Avoidance → FoMO → Anxiety	0.03^**^	0.01	0.01	0.06	16.84%
Loneliness →FoMO → Anxiety	0.11^***^	0.02	0.07	0.16	32.11%
Positive Solitude → FoMO → Stress	0.00	0.01	0.00	0.02	-
Eccentricity → FoMO → Stress	0.00	0.01	-0.01	0.01	-
Social Avoidance → FoMO → Stress	0.02^*^	0.01	0.01	0.04	18.81%
Loneliness → FoMO → Stress	0.07^**^	0.02	0.02	0.11	20.06%

FoMO: fear of missing out. *β*: standardized indirect effects, *SE*: standard error.

^*^p < 0.05, ^**^p < 0.01, ^***^p < 0.001.

Bootstrap method with 5000 bootstrap samples was used to compute the 95% confidence intervals.

## Discussion

The current study explored the relationship between solitude behaviors, FoMO, and mental health. Revealing the mechanism of the role of FoMO under the influence of solitude behavior, which had important implications for the promotion of mental health.

Different associations among four types of solitude behaviors were explored in this study. The results indicated that positive solitude was positively associated with eccentricity, and social avoidance was positively associated with loneliness. These results were consistent with previous research suggesting that positive solitude and eccentricity were self-determined solitude; social avoidance and loneliness were non-self-determined solitude [16]. Consistent with previous literature [28], our findings also observed eccentricity as a positive predictor of social avoidance. It may be that although eccentricity was a self-determining attribute, it was frequently one of the most prominent pre-morbid characteristics of schizophrenia, involving negative aspects [29]. People who exhibited this tendency frequently had trouble maintaining healthy relationships with others, preferred to live alone, and often refused to talk with others [16]. Eccentricity was thought to reflect both a lack of interest in engagement and the active avoidance of social interaction opportunities. Eccentricity and social avoidance frequently removed them from opportunities to engage in social interaction, and mainly reflected the negative sides of psychological characteristics. According to previous studies, a form of active social isolation called hikikomori has been frequently combined with avoidance of social interactions and withdrawal behavior [30, 31]. Furthermore, the results showed that positive solitude was negatively associated with loneliness. The positive solitude satisfied higher psychological needs (e.g., self-improvement, meditation) and received positive emotional experiences in the solitary state; the loneliness was dissatisfied with the current stage of interpersonal relationships and desired to establish interpersonal relationships with others, which led to interpersonal needs, so it was logical to understand that the negative association between them [32, 33].

Noteworthily, the current study found that social avoidance and loneliness, non-self-determined solitude, predicted mental health through FoMO, which was consistent with the hypothesis. While positive solitude and eccentricity, self-determined solitude, did not predict mental health through FoMO. Additionally, the results still held in the model with depression, anxiety, and stress as dependent variables. This observation hinted at an important point that social avoidance was essentially the fear of ridicule and embarrassment caused by making mistakes in interpersonal interactions [34]. Despite a desire to engage with others, this temperamental trait caused social withdrawal due to social fears. There were some previous studies suggest that social avoidance has been linked to unpleasant experiences [35, 36]. To alleviate this negative emotion, individuals tended to avoid interacting with others. Loneliness was a negative experience that arose when individuals were alone and were dissatisfied with the quality or quantity of social relationships [37]. Whether social avoidance or loneliness was dominant when their relatedness needs were not met, individuals still tended to internally approach and integrated with people, which in turn triggered the FoMO and harmed people’s mental health. Conversely, the need for relatedness was low when actively choosing to be alone and therefore did not lead to FoMO and accompanying mental health problems. Results from the previous study indicated that self-determined solitude demonstrated a positive correlation with personal growth and self-acceptance, whereas not self-determined solitude showed negative correlations with these desirable outcome variables [38]. There was evidence suggesting that FoMO was related to less satisfaction with basic psychological needs, which was frequently connected to the emergence of internalizing issues [9]. It may be that self-determined solitude are more likely comfortable with solitude, allowing them to facilitate the basic human needs of relatedness, instead of serving to promote FoMO. Nguyen et al. found that self-determined solitude not only enhanced subjective well-being but also led to relaxation and stress relief [39]. Nelson also argued that while unsociable individuals showed greater levels of depression, relationships had fewer problems [40].

Although both social avoidance and loneliness predicted depression, anxiety, and stress through FoMO, some differences remained. And the results of the path showed that while social avoidance did not directly predict depression, it did directly predict stress and anxiety. Perhaps social avoidance is an avoidance type of anxiety that results from being nervous about social interaction [8]. Moreover, FoMO consistently played a partial mediation role in the association of loneliness with depression, anxiety, and stress. There may be psychological mechanisms other than FoMO in predicting loneliness on mental health. For example, Domènech-Abella et al. noted that among those who felt lonely, individuals with depression had a smaller social network [41]. Therefore, speculation on the relationship between the two needs to be further validated and explored in future studies.

As our model suggests, FoMO seemed to undermine mental health benefits, which might interfere with the quality of their psychological adjustment and daily life. FoMO’s consequences could have negative long-term impacts on societal communication and well-being [42]. Effective interventions may need to concentrate on how non-self-determined solitude fuel such FoMO concerns. FoMO is a concept that explores the fear of social exclusion. Strengthening social connections and supports can both help individuals escape feelings of disconnectedness and offer opportunities for connecting with other people without having the feeling of missing out on something. Then the reduction of such negative experiences in turn improves mental health [43, 44]. It is potential for us to be concerned about individuals’ experiences when interacting with their peers, in order to preempt their dissatisfaction, which has been linked to detrimental effects on FoMO, and subsequently created psychological disorders [42]. There were some limitations in this study. Aside from the solitude behaviors themselves, the personality characteristics of the samples need to be taken into account when examining the link between solitude behaviors and FoMO [45, 46]. Additionally, this study was a cross-sectional study, and future studies may consider using longitudinal studies to further validate our findings. It also would be important for future investigations to examine whether the relationship between self-determined solitude and mental health is moderated by other psychological functioning.

## Conclusions

The roles of solitude behaviors in the relationship between FoMO and mental health differed. Social avoidance and loneliness affected mental health through the FoMO, whereas positive solitude and eccentricity did not. This study provided opportunities for researchers to learn about the possible factors that trigger mental health problems and revealed the mechanisms by which FoMO acted under the influence of solitude behaviors, with important implications for the promotion of mental health.

## Data Availability

The datasets used and/or analysed during the current study is available from the corresponding author on reasonable request.
